# Background parenchymal enhancement in contrast-enhanced MR imaging suggests systemic effects of intrauterine contraceptive devices

**DOI:** 10.1007/s00330-022-08809-0

**Published:** 2022-05-07

**Authors:** Luisa Charlotte Huck, Daniel Truhn, Caroline Wilpert, Eloisa Zanderigo, Vanessa Raaff, Ebba Dethlefsen, Maike Bode, Christiane Katharina Kuhl

**Affiliations:** Department of Diagnostic and Interventional Radiology, University of Aachen, RWTH, Pauwelsstr. 30, 52074 Aachen, Germany

**Keywords:** Breast MRI, Levonorgestrel, Levonorgestrel-releasing intrauterine device, Contraception, Background parenchymal enhancement

## Abstract

**Objectives:**

Levonorgestrel-releasing intrauterine contraceptive devices (LNG-IUDs) are designed to exhibit only local hormonal effects. There is an ongoing debate on whether LNG-IUDs can have side effects similar to systemic hormonal medication. Benign background parenchymal enhancement (BPE) in dynamic contrast–enhanced (DCE) MRI has been established as a sensitive marker of hormonal stimulation of the breast. We investigated the association between LNG-IUD use and BPE in breast MRI to further explore possible systemic effects of LNG-IUDs.

**Methods:**

Our hospital database was searched to identify premenopausal women without personal history of breast cancer, oophorectomy, and hormone replacement or antihormone therapy, who had undergone standardized DCE breast MRI at least twice, once with and without an LNG-IUD in place. To avoid confounding aging-related effects on BPE, half of included women had their first MRI without, the other half with, LNG-IUD in place. Degree of BPE was analyzed according to the ACR categories. Wilcoxon-matched-pairs signed-rank test was used to compare the distribution of ACR categories with vs. without LNG-IUD.

**Results:**

Forty-eight women (mean age, 46 years) were included. In 24/48 women (50% [95% CI: 35.9–64.1%]), ACR categories did not change with vs. without LNG-IUDs. In 23/48 women (48% [33.9–62.1%]), the ACR category was higher with vs. without LNG-IUDs; in 1/48 (2% [0–6%]), the ACR category was lower with vs. without LNG-IUDs. The change of ACR category depending on the presence or absence of an LNG-IUD proved highly significant (*p* < 0.001).

**Conclusion:**

The use of an LNG-IUD can be associated with increased BPE in breast MRI, providing further evidence that LNG-IUDs do have systemic effects.

**Key Points:**

• *The use of levonorgestrel-releasing intrauterine contraceptive devices is associated with increased background parenchymal enhancement in breast MRI.*

• *This suggests that hormonal effects of these devices are not only confined to the uterine cavity, but may be systemic.*

• *Potential systemic effects of levonorgestrel-releasing intrauterine contraceptive devices should therefore be considered.*

## Introduction

Intrauterine contraceptive devices (IUD) are used by more than 168 million women worldwide and are the most common method of reversible female contraception [1]. Between 2009 and 2012, the utilization of levonorgestrel-releasing intrauterine devices (LNG-IUDs) increased from 8.5 to 11.6% among women who use contraception in the USA [2]. There are two fundamentally different LNG-IUDs: One class of LNG-IUDs releases copper ions that appear to interfere with nidation and sperm motility; the other class is levonorgestrel-releasing intrauterine devices (LNG-IUD) [1]. It has been argued that LNG-IUDs act as a local source of progestogen on the uterus and are not associated with a systemic hormonal effect [1, 3]; in line with this, the Centers for Disease Control and Prevention distinguish local intrauterine methods (copper or LNG-IUDs) from hormonal methods of contraception [4]. However, there is emerging evidence that women with LNG-IUDs experience similar side effects compared to women on oral or percutaneous hormonal medication, including mood changes as well as an increased risk of breast cancer, especially for women over 50 [5–8]. A recent study showed that in women with LNG-IUDs, there are lower, but detectable, levels of levonorgestrel within the breasts of women using LNG-IUDs [9], and there is a case report on a woman whose breast density increased markedly after using LNG-IUD [10].

Background parenchymal enhancement (BPE) in contrast-enhanced MR imaging is known to be a sensitive marker for the level of hormonal stimulation of the breast [11, 12]. BPE is independent of mammographic breast density and the amount of fibroglandular tissue in breast MRI [13]. It is a manifestation of systemic physiologic processes which is usually symmetric. BPE is stronger in pre- vs. postmenopausal women [11, 12] and varies across the menstrual cycle [14, 15]. BPE increases with hormone replacement therapy (HRT) [12], and is reduced by anti-hormonal therapy, or by factors that reduce endogenous hormone production (e.g., systemic chemotherapy), or by reducing the glandular, i.e., hormone-sensitive components of the breast, e.g., by radiotherapy [16–20]. A high degree of BPE may reduce the sensitivity and specificity of breast MRI and has been established as an independent factor of breast cancer risk [21–24].

We therefore investigated the association between LNG-IUD use and BPE in breast MRI. We used BPE as an established imaging biomarker for hormonal stimulation of the breast parenchyma, to further explore possible systemic effects of LNG-IUDs.

## Materials and methods

### Inclusion criteria

This retrospective study was approved by the local institutional review board (EK 208/21). Written informed consent was waived by the institutional review board.

We included premenopausal women who underwent breast MRI in our department for clinical indications at least twice: at least once with and at least once without an LNG-IUD in place. In order to prevent the effects of normal aging from confounding our results, we took care to include as many women who had their first MRI with LNG-IUD, and their follow-up MRI after LNG-IUD removal (group A) as we included women who had their first MRI without LNG-IUD, and their follow-up MRI after LNG-IUD placement (group B). In accordance with the institution’s internal practice guidelines, all pre-menopausal women undergoing MRI screening or problem solving are scheduled for the 2^nd^ week of the menstrual cycle; however, women with LNG-IUDs may not experience a regular cycle any more.

Exclusion criteria were as follows: a personal history of breast cancer, including staging examinations for a recent diagnosis of breast cancer; a history of radiation therapy, of chemotherapy, or of oophorectomy; use of other types of hormonal or preventive anti-hormonal treatment; or use of additional oral contraceptive medication.

Information on presence of LNG-IUD was collected by the radiological technologist at the time of the MRI study and recorded in the respective women’s electronic medical record as part of routine MRI safety precautions. Since safety precautions differ for copper-releasing LNG-IUDs vs. nonmetallic LNG-IUDs, the type of LNG-IUD is also recorded. The time of LNG-IUD placement was not consistently recorded. At the time this data was collected, there were two different LNG-IUDs available in Germany, one releasing 20 mg levonorgestrel per 24 h (Mirena®, Bayer) or 13.5 mg per 24 h (Jaydess®, Bayer). Based on the manufacturer’s information, the contraceptive effect lasts for about 3 (Jaydess®) or 5 (Mirena®) years, with gradual decay of the released amount of hormones. Current guidelines mandate to either remove or replace IUDs beyond the recommended time frame.

### Breast MR imaging technique

Contrast–enhanced bilateral breast MRI was performed according to a previously published standardized protocol [25] on a 1.5-T system (Achieva, Philips Medical System) with a multi-channel breast coil (Invivo). In short, the protocol consists of an axial bilateral T2-weighted fast spin-echo and an axial bilateral dynamic series consisting of five dynamic phases (repetition time/echo time [TR/TE]: 240/4.6, flip angle: 90°) before and four times after bolus injection of 0.1 mmol/kg body weight (Gadovist, Bayer Healthcare). Image subtraction was performed at all post-contrast phases. T2-weighted fast spin-echo images were part of the clinical breast MRI protocol but were not included for analysis within this study.

### Breast MRI interpretation and classification of BPE

Breast MRI studies were prospectively read by two different experienced breast radiologists with 25 and 8 years of experience, respectively, on a Picture Archiving and Communication System (PACS) workstation (Philips IntelliSpace). For each breast MRI examination, BPE was determined as part of clinical case reading and followed closely defined institutional standards. All image interpretation was done blinded to the fact whether LNG-IUDs were present or absent. According to the Breast Imaging Reporting and Data System (BI-RADS) guidelines, BPE was assessed in the first contrast-enhanced dynamic series (acquired 70 s after contrast injection) and was categorized as “minimal” (MR-ACR I), “mild (MR-ACR II), “moderate” (MR-ACR III), and “marked” (MR-ACR IV). If the BPE was asymmetric, the breast with the highest MR-ACR category was considered. Nonsubtracted images were used for assessment in case motion artifacts degraded the subtraction quality.

### Data analysis

**Fig. 1:**
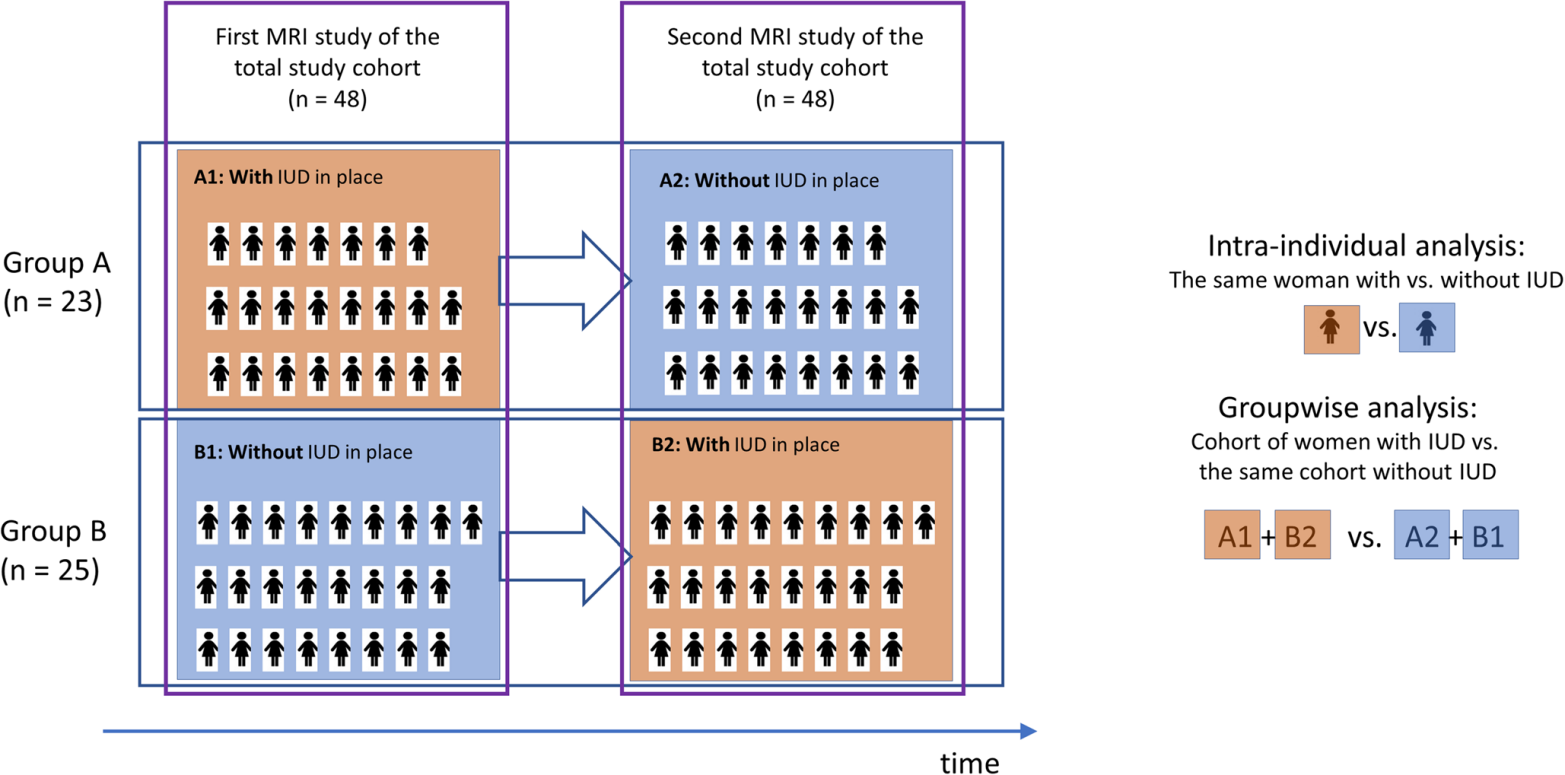
Study design. The total study cohort consisted of 48 premenopausal women. Women underwent their first MRI study (left column) either with an IUD in place (group A, examination A1) or without an IUD in place (group B, examination B1). At the second MRI study (right column), women of group A had the MRI after IUD removal (examination A2); women of group B had the MRI after IUD placement (examination B2). The main comparison is on the respective levels of BPE in the MRI with vs. without LNG-IUD for each individual woman. In addition, a groupwise analysis of BPE in women with (A1 and B2) vs. without (A2 and B1) LNG-IUD was done. A1, first MRI examination of women of group A (with LNG-IUD in place); A2, second MRI examination of women of group A (without LNG-IUD in place); B1, first MRI examination of women of group B (without LNG-IUD in place); B2, second MRI examination of women of group B (with LNG-IUD in place)

The type of analyses is described in Fig. 1. The main comparison is on the respective levels of BPE, categorized according to the MRI-ACR categories, intra-individually in the MRI with vs. without LNG-IUD of each individual woman. In addition, a groupwise analysis of BPE in women with (examination A1 and B2) vs. without (examinations A2 and B1) LNG-IUD was done. Since the date of IUD placement was not known in all women, we re-did both analyses (groupwise and intra-individual) restricted to the subcohort of women whose date of IUD placement was known to be within the respective therapeutic range, i.e., 5 years after placement for Mirena and 3 years after placement for Jaydess.

### Statistical analysis

MR-ACR BPE categories of the same women depending on presence or absence of an LNG-IUD were analyzed using the Wilcoxon-matched pairs signed-rank test. SPSS software version 27.0 (IBM) was used for statistical analysis. A *p* value less than 0.05 was considered statistically significant.

## Results

### Study cohort

**Fig. 2:**
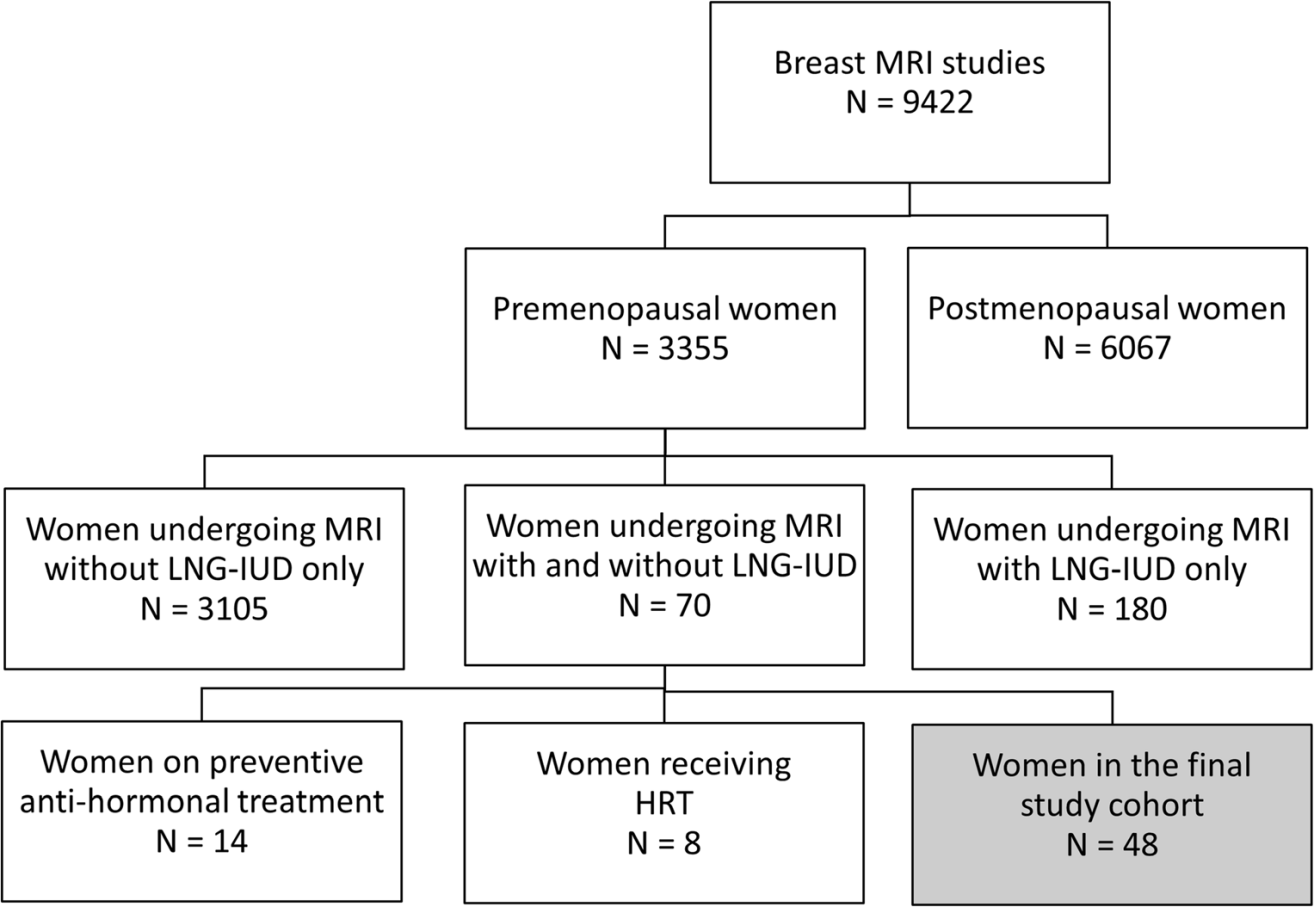
Flow chart of women examined in our institution between 2014 to 2020. LNG-IUD = levonorgestrel-releasing intrauterine contraceptive device, HRT = hormone replacement therapy

Between 2014 and 2020, a total of 9422 women underwent breast MRIs in our institution. Of these, 3355 were premenopausal. A total of 250/3355 women were known to use an LNG-IUD. Of these, a total of 70 had undergone breast MRI without and with the LNG-IUD in place. Of these, 22 women had to be excluded because they were currently receiving hormonal treatment, 8 women because they were on HRT or oral contraceptives in addition to the LNG-IUD, and 14 for being on preventive anti-hormonal treatment. Accordingly, the final study cohort consisted of 48 women (mean age, 46 years ± 6.2) (Fig. 2). Clinical indications for breast MRI for the respective women were mostly routine screening, and less often problem solving (i.e., follow-up after MR-BI-RADS 3 lesion, equivocal sonographic findings, equivocal mammographic findings, and palpable breast lesion). By study inclusion criteria, we did not include women with a recent diagnosis of breast cancer; i.e., we did not include women who underwent MRI for staging or response assessment.

The majority of women (46/48) of the final cohort had a Mirena® LNG-IUD in place; the remaining two had the Jaydess® device in place.

As per study inclusion criteria, half of the women (48%, 23/48; group A) had their first MRI done with an LNG-IUD in place (A1), and the second MRI after IUD removal (A2); the other half (52%, 25/48; group B) had their first MRI done without an LNG-IUD (B1), and their second MRI after LNG-IUD placement (B2) (Fig. 1). With this, the age distribution of women at the time of their respective MRI examination with IUD was identical to the age of women at the time of their respective MRI examination without IUD. Because women happened to be evenly distributed in the analysis cohort, no patient had to be excluded to avoid effects of normal aging. The median time between the two MRI examinations was 26 months (range 1–77 months) (Tables 1 and 2).

**Table 1:** Demographic details of 48 study participants at the time of MRI with and without levonorgestrel-releasing intrauterine contraceptive devices (LNG-IUD) in place

	Women undergoing MRI without LNG-IUD		Women undergoing MRI with LNG-IUD	
Age				
Mean ± SD	45 ± 7.3		46 ± 6.2	
Range	23–56		28–56	
Time between MRIs (months)				
Mean ± SD	26 ± 14.1			
Range	1–77			
Clinical indication to undergo breast MRI	*n*	%	*n*	%
Routine screening	39	81	34	71
Follow-up after MR-BI-RADS 3 lesion	5	11	3	6
Equivocal sonographic findings	4	8	6	13
Equivocal mammographic findings	0	0	3	6
Palpable breast lesion	0	0	2	4
IUD			*n*	%
Mirena			46	96
Jaydess			2	4

**Table 2:** Demographic details of 48 study participants, by study subcohort

	Group A		Group B	
	First MRI with IUD Second MRI without IUD (*n* = 23)		First MRI without IUD Second MRI with IUD (*n* = 25)	
Time between the two MRI studies (months)				
Mean ± SD	23 ± 15.1		28 ± 13.0	
Range	1–77		11–57	
Type of IUD	*n*	%	*n*	%
Mirena	22	96	24	96
Jaydess	1	4	1	4

The date of IUD placement was known in 25 women at the time of their MRI study with IUD in place, and was within the recommended time frame (5 years for Mirena, 3 years for Jaydess) in these 25 patients. Age distribution (mean 45 years ± 7.2, range 24–56) and the respective clinical indications to undergo MRI (screening vs. problem solving) of the women of this subgroup matched with the age distribution of the entire cohort.

### Visual analysis of BPE

**Fig. 3:**
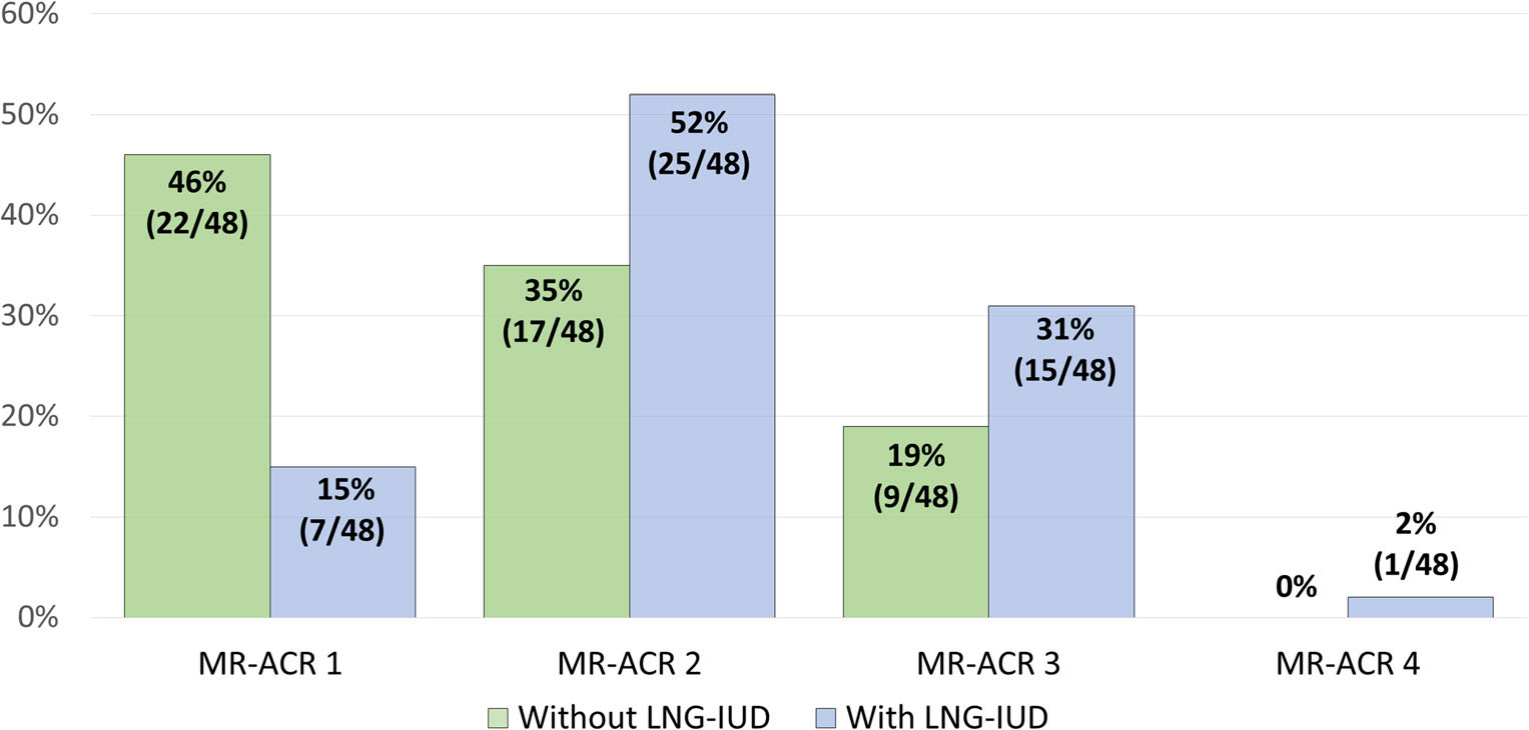
Distribution of background parenchymal enhancement MR-ACR categories depending on the presence or absence of a levonorgestrel-releasing intrauterine contraceptive device (LNG-IUD) in the entire study cohort

At a groupwise analysis (grouping women with LNG-IUD vs. women without LNG-IUD), the following distribution of MR-ACR categories was observed: Without the LNG-IUD, the distribution of MR-ACR categories 1, 2, 3, and 4 was 46% (22/48), 35% (17/48), 19% (9/48), and 0%, respectively. With the LNG-IUD, the respective distribution was 15% (7/48), 52% (25/48), 31% (15/48), and 2% (1/38) (Fig. 3).

**Fig. 4:**
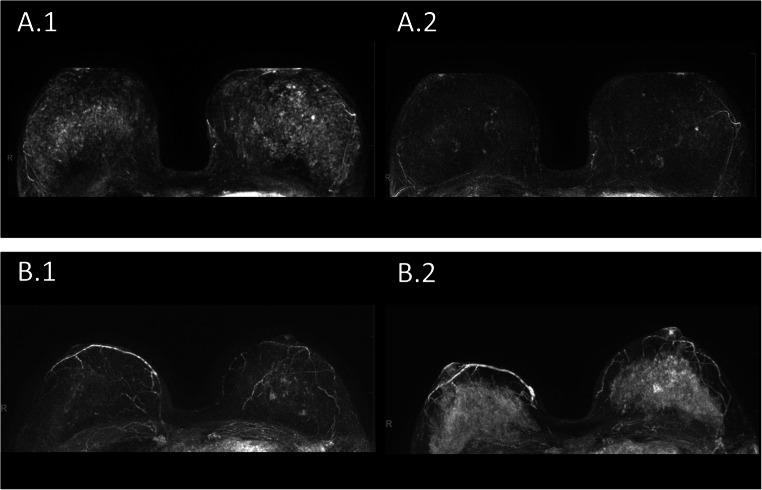
BPE on DCE-MRI in women with vs. without LNG-IUD. **A.1** and **A.2**: a premenopausal woman undergoing MRI for screening because of dense breast tissue of group A. Maximum-intensity projection images (MIPs) of the first post-contrast subtracted images of the woman’s MRI (**A.1**) at age 44, with LNG-IUD (Mirena®) in place since 3 years, and (**A.2**) the same woman, now aged 47, after LNG-IUD removal. BPE was categorized as MR-ACR 3 in A.1 and MR-ACR 2 in A.2. **B.1** and **B.2**: a premenopausal woman undergoing MRI for screening because of dense breast tissue of group B. Maximum-intensity projection images (MIPs) of the first post-contrast subtracted images of the woman’s MRI (**B.1**) at age 41, without LNG-IUD in place, and (**B.2**) the same woman, now aged 42, after LNG-IUD placement (Mirena®) 1.5 years ago. BPE was categorized as MR-ACR 2 in B.1 and MR-ACR 4 in B.2

At an intra-individual analysis (comparing the MR-ACR category of women without vs. that of the same women with LNG-IUD), the following findings were made: In 23/48 women (48%), LNG-IUD was associated with higher BPE; in 1/48 (2%), with lower BPE; and in 24/48 (50%), with similar BPE as indicated by the respective ACR categories (Fig. 4).

Among the 23/48 women (48%) with an increase in MR-ACR categories, the difference was by one category in most women (22/23), by two categories in 1/23; a deviation by three categories was not observed. The change of MR-ACR category with vs. without LNG-IUD was highly significant (*p* < 0.001).

When restricting the analysis to the subcohort of 25 women whose date of IUD placement was known, and thus were definitely known to have IUDs that release LNG in therapeutic levels, similar results were observed as in the entire cohort: MR-ACR categories were higher with LNG-IUD in place in 14 of 25 women (56%), with an increase by one MR-ACR category in 13/25 and an increase by two MR-ACR categories in 1/25 compared to the same women’s MRI study without LNG-IUD; the remaining 11 women (11/25, 44%) showed similar BPE. Also, in this subcohort, the distribution of MR-ACR categories of MRI in women without vs. with LNG-IUD proved statistically significantly different (*p* < 0.001) (Table 3).

**Table 3:** Distribution of background parenchymal enhancement MR-ACR categories depending on presence or absence of a levonorgestrel-releasing intrauterine contraceptive device (LNG-IUD), in the entire cohort and the cohort of women whose date of LNG-IUD placement was known, and was within the 3- or 5-year time window of therapeutically relevant local LNG release

MR-ACR category	Entire cohort (*n* = 48)				Subgroup of women whose date of IUD placement was known, and was within the recommended time window			
	Without IUD		With IUD		Without IUD		With IUD	
	*n*	%	*n*	%	*n*	%	*n*	%
ACR 1	22	46	7	15	13	52	5	20
ACR 2	17	35	25	52	11	44	12	48
ACR 3	9	19	15	31	1	4	8	32
ACR 4	0	0	1	2	0	0	0	0

## Discussion

In this intra-individual study of serial breast MRI in premenopausal healthy women, we observed that the presence of a levonorgestrel-releasing intrauterine device (LNG-IUD) is associated with an increase in background parenchymal enhancement: background parenchymal enhancement rates observed in women with an LNG-IUD in place were significantly higher than in the same women without LNG-IUD. Removing an intrauterine device was associated with a reduction in background parenchymal enhancement; insertion of an intrauterine device was associated with an increase. This suggests that LNG-IUDs—although designed to provide a local source of hormone replacement only—do also have systemic effects that are similar to those of oral or percutaneous HRT.

The distribution of BPE of women in our cohort without LNG-IUD was in good agreement with published values for premenopausal women [13], with 79% of women exhibiting an MR-ACR A or B, and 21% an MR-ACR-category C, and none to a category D. However, with an LNG-IUD in place, the same women’s distribution of MR-ACR categories was shifted toward higher categories, with 15% belonging to MR-ACR category 1, 52% to MR-ACR category 2, 31% to MR-ACR category 3, and 2% to MR-ACR category 4.

On an intra-individual level, we observed that in about half of women (48%), BPE of the same women was higher with vs. without an LNG-IUD in place, whereas in the other half, BPE did not change. Still, the change of ACR category depending on presence or absence of an LNG-IUD proved highly significant (*p* < 0.001).

In the majority of women, the normal fibroglandular tissue exhibits only mild enhancement, occurring only during the late phase of the dynamic series. However, as the glandular components of the normal breast respond to hormonal stimulation, and since hormonal stimulation just as the volume fraction of milk glands vs. fibrous components of fibroglandular tissue varies between women, there is a broad range of enhancement kinetics of the normal fibroglandular tissue referred to as “background parenchymal enhancement” [26–28].

Changes in BPE can be attributable to a variety of factors that modulate the level of hormonal stimulation of the breast, or the amount of glandular vs. fibrous components of BPE. There are endogenous effects, i.e., variable hormonal stimulation due to normal aging [29, 30], and exogeneous effects such as HRT, anti-hormonal treatment, resection of ovaries, radiotherapy, and chemotherapy [16, 17, 20, 31]. To exclude such exogenous hormonal effects to confound our results, we included healthy women without history of cancer treatment, and without HRT or preventive anti-hormonal therapy. To exclude aging-related effects as a confounding factor, we took care that half of the women included in our cohort had undergone their first MRI study without LNG-IUD, and the second after LNG-IUD placement, and the other half vice versa.

The fact that an increased BPE was only observed in about half of the women with LNG-IUD in place, whereas BPE was unchanged in the other half, is noteworthy, and there are several possible explanations: For one, although we used an intra-individual study design, and a study design that should avoid confounding aging-related effects, endogenous hormone levels may differ in the same woman across different points in time. Differences in hormonal metabolism, and genetic and epigenetic factors, may play a role and could lead to heterogeneity of “BPE response” to LNG-IUD in the study cohort [27, 28]. However, if variable hormonal metabolism drove the observed results, then we would expect to see random effects, which would mean that we would expect to see also cases with lower BPE in women with LNG-IUD. Yet this was not the case: In women with definitely hormone-releasing IUDs, BPE was either stronger or unchanged compared to the same women’s BPE without LNG-IUD—but not lower.

Another explanation for the lack of association between LNG-IUD and BPE observed in about half of women could be the fact that hormone release by LNG-IUDs decreases over time; the two LNG-IUDs used in our cohort have a therapeutic window of 3 and 5 years, respectively [1, 32, 33]. Since this is a retrospective analysis, we could not control the time interval between LNG-IUD placement and the MRI study; in almost half of the women, the exact date of LNG-IUD placement was not known. Current guidelines mandate the removal or replacement of LNG-IUDs after the recommended time, and all 25 women for whom the date of LNG-IUD placement was known had their LNG-IUDs placed within the recommended time. Still, it is conceivable that among the remaining 23 women whose date of LNG-IUD placement was not known could have had an LNG-IUD that was already beyond the recommended time, possibly releasing LNG below the therapeutic level—which could explain the observed “no-change” of BPE with vs. without LNG-IUD. To further investigate this, we did an analysis restricted to the subcohort of women for whom the date of IUD placement was known to be within the therapeutic time range. Here, the same results were observed as in the entire cohort, with half of women exhibiting stronger BPE on their MRI with LNG-IUD, whereas the other half did not exhibit a change of BPE. This suggests that a possible decay of LNG-IUD hormonal release does not explain the fact that BPE is unchanged in about 50% of women.

Finally, an explanation why the MR-ACR categories were unchanged in half of the women could be attributable to the broadness of the ACR BPE categories per se that may not be sufficient to distinguish mild changes of BPE.

Several studies demonstrated that BPE varies between the menstrual cycle and it is known that the 2^nd^ week of the menstrual cycle is an optimal period for breast MRI with the lowest level of BPE [14, 26, 34]. Therefore, in accordance with the institution’s internal practice guidelines, all pre-menopausal women undergoing MRI screening or problem solving are scheduled for the 2^nd^ week of the menstrual cycle. However, one in five women no longer has a regular menstrual cycle after insertion of an LNG-IUD [35], which may have resulted in a few women not being examined during the 2^nd^ week of their menstrual cycle. Although variable BPE may not necessarily confound the diagnostic accuracy of breast MRI interpretation [36, 37], it would confound results where BPE is used as a metric for systemic hormonal effects.

The shift of BPE distribution observed under LNG-IUD was similar to that published for postmenopausal women under oral or percutaneous HRT [31, 38, 39]. This is remarkable because here, we included pre-menopausal women who will have a higher baseline (i.e., pre-LNG-IUD placement) BPE level even before LNG-IUD placement than postmenopausal women who are put on HRT.

Our results have clinical implications. From a diagnostic point of view, the increase of BPE observed in women on LNG-IUDs was mostly mild. Still, since cancer detection, especially DCIS detection in breast MRI depends on the radiologists’ ability to identify significant enhancement, our results indicate that radiologists should at least be aware of the possibility of higher BPE levels in women having LNG-IUDs [22, 40]. A clinically possibly more important implication of our findings is the actual association between LNG-IUDs and BPE. The fact that presence of an LNG-IUD is associated with stronger BPE, and that the effects of LNG-IUD and oral or percutaneous hormone replacement appear to be similar, is another important evidence for the fact that LNG-IUDs are indeed associated with systemic hormonal stimulation similar to that observed in women put on systemic HRT [39]. This is relevant in view of the ongoing debate on whether or not the very low serum concentrations of levonorgestrel that are measured in women with LNG-IUD can cause systemic side effects. Several studies have suggested a higher rate of depression, abdominal pain, weight gain, and lack of sexual interest in women on LNG-IUD [6–8].

Our study has limitations. The most important being the small cohort size, which was in part due to the strict selection criteria used for this study; however, these selection criteria enabled us to study the possible effects of LNG-IUDs by an intra-individual design, and by excluding all possible confounding factors, including age-related factors that could confound serial MRI studies. Another limitation is the fact that we do not know the exact date of LNG-IUD placement in about half of the women. However, since the level of hormone release decreases with longer LNG-IUD use, this implies that our study might underestimate, but not overestimate, the effects of LNG-IUD on BPE. Moreover, to account for this, we did a subgroup analysis restricted to women whose LNG-IUDs were known and thus known to be within the therapeutic range. Moreover, we did not determine the serum levonorgestrel levels, which would be an important goal of further prospective studies on this issue. In addition, in this study, we did not investigate how breast density changes with an LNG-IUD in place. Finally, all but two women in our cohort had the same type of LNG-IUD (Mirena®), reflecting the current market share of this device in our country, and too small a cohort to allow a meaningful subgroup analysis stratified by subtype of LNG-IUD. Results in the two women who had the Jaydess® device were similar to those observed in women carrying the Mirena device, with one woman experiencing stronger BPE, the other no change of BPE in the MRI with vs. without LNG-IUD.

In conclusion, our results indicate that LNG-IUDs—i.e., devices expected to exert a purely local hormonal effect on the uterus—are indeed associated with increased BPE, as an established marker of hormonal stimulation of the breast. Accordingly, there appear to be systemic (in-breast) effects of LNG-IUDs, i.e., BPE, similar to those associated with oral or percutaneous hormonal therapies. For radiologists interpreting breast MRI studies, knowledge about LNG-IUD use as a cause of BPE may be important.

## Abbreviations

BI-RADS: Breast imaging reporting and data system
BPE: Background parenchymal enhancement
HRT: Hormone replacement therapy
IUD: Intrauterine contraceptive devices
LNG-IUD: Levonorgestrel-releasing intrauterine device
PACS: Picture Archiving and Communication System

## Declarations

### Guarantor

The scientific guarantor of this publication is the last author of this manuscript.

### Statistics and biometry

No complex statistical methods were necessary for this paper.

### Methodology

• retrospective

• observational

• performed at one institution
